# Supplementary material for: New insights into the impact of financial inclusion on economic growth: A global perspective
Source: PLoS One. 2022 Nov 17;17(11):e0277730. doi: 10.1371/journal.pone.0277730 (PMC9671310; doi:10.1371/journal.pone.0277730)
Supplement: S1 Appendix — (DOCX) [file pone.0277730.s001.docx]

**Appendix A**

**Table A1** CFII aggregate index.

| Dimension | Indicators | Full panel | Income level panel: Allocated weights | | | | | |
| --- | --- | --- | --- | --- | --- | --- | --- | --- |
|  |  |  | Low-income | Middle-income | Upper middle-income | High-income | OECD | Non-OECD |
| Penetration | 1. Number of deposit accounts per 1,000 adults 2. Number of depositors per 1,000 adults | 0.70  0.30 | 0.60  0.40 | 0.70  0.30 | 0.70  0.30 | 0.70  0.30 | 0.70  0.30 | 0.70  0.30 |
| Availability | 1. Number of banks per 100,000 adults 2. Number of ATMs per 100,000 adults | 0.50  0.50 | 0.50  0.50 | 0.50  0.50 | 0.50  0.50 | 0.50  0.50 | 0.50  0.50 | 0.50  0.50 |
| Usage | 1. Number of loan accounts in banks per 1,000 adults 2. Number of borrowers from banks per 1,000 adults | 0.70  0.30 | 0.70  0.30 | 0.70  0.30 | 0.70  0.30 | 0.70  0.30 | 0.70  0.30 | 0.70  0.30 |
| Dimension | Indicators | Regional panel: Allocated weights | | | | | | |
|  |  | East Asia and Pacific | Europe and Central Asia | Latin America and Caribbean | MENA | North America | South Asia | Sub-Saharan Africa |
| Penetration | 1. Number of deposit accounts per 1,000 adults 2. Number of depositors per 1,000 adults | 0.70  0.30 | 0.70  0.30 | 0.60  0.40 | 0.70  0.30 | 0.60  0.40 | 0.60  0.30 | 0.70  0.30 |
| Availability | 1. Number of banks per 100,000 adults 2. Number of ATMs per 100,000 adults | 0.50  0.50 | 0.50  0.50 | 0.50  0.50 | 0.50  0.50 | 0.50  0.50 | 0.50  0.50 | 0.50  0.50 |
| Usage | 1. Number of loan accounts in banks per 1,000 adults 2. Number of borrowers from banks per 1,000 adults | 0.70  0.30 | 0.70  0.30 | 0.70  0.30 | 0.70  0.30 | 0.70  0.30 | 0.70  0.30 | 0.70  0.30 |
| Notes: ATM = Automated teller machine, OECD = Organization for Economic Cooperation and Development, MENA = Middle-East and North Africa. The weights are allocated using the means values of the indicators. For instance, the weight of banking penetration for the full panel is estimated as the number of deposit accounts to be $667/1000\approx0.70,$while its weight for the number of depositors would be $302/1000\approx0.30,$and so on (see, for instance, Sarma, 2008). Thus, the allocated weights are rounded to the nearest absolute integers. | | | | | | | | |
