# Supplementary material for: New insights into the impact of financial inclusion on economic growth: A global perspective
Source: PLoS One. 2022 Nov 17;17(11):e0277730. doi: 10.1371/journal.pone.0277730 (PMC9671310; doi:10.1371/journal.pone.0277730)
Supplement: S2 Appendix — (DOCX) [file pone.0277730.s002.docx]

**Appendix B**

**Table B1** Descriptive statistics.

| Panels |  | | | |  | | | |  | | | |  | | | |  | | | |
| --- | --- | --- | --- | --- | --- | --- | --- | --- | --- | --- | --- | --- | --- | --- | --- | --- | --- | --- | --- | --- |
|  | Mean | Min. | Max. | Std. dev. | Mean | Min. | Max. | Std. dev. | Mean | Min. | Max. | Std. dev. | Mean | Min. | Max. | Std. dev. | Mean | Min. | Max. | Std. dev. |
| **Full panel** | 2.74 | -3.27 | 4.53 | 1.95 | 0.69 | 0.26 | 0.89 | 0.03 | 55.19 | 53.98 | 58.82 | 1.44 | 38.42 | 52.12 | 23.19 | 5.29 | 2.78 | 1.05 | 5.32 | 1.02 |
| **Income level economies** | | | | | | | | | | | | | | | | | | | | |
| Low-income | 2.00 | -4.10 | 6.77 | 3.26 | 0.78 | 0.38 | 0.91 | 0.04 | 88.71 | 85.85 | 91.21 | 1.93 | 12.42 | 10.87 | 16.30 | 1.67 | 3.90 | 2.79 | 5.77 | 1.04 |
| Middle-income | 5.48 | -1.28 | 8.91 | 2.25 | 0.73 | 0.41 | 0.88 | 0.10 | 52.79 | 50.93 | 58.00 | 2.19 | 77.68 | 55.02 | 78.52 | 9.97 | 5.48 | 1.28 | 8.91 | 1.53 |
| Upper middle-income | 5.69 | -0.59 | 9.64 | 2.39 | 0.69 | 0.36 | 0.93 | 0.08 | 43.73 | 41.96 | 48.37 | 1.81 | 90.44 | 63.58 | 93.92 | 4.97 | 2.77 | 1.69 | 4.13 | 0.72 |
| High-income | 6.13 | -4.88 | 11.07 | 2.77 | 0.71 | 0.50 | 0.83 | 0.03 | 40.09 | 38.77 | 42.18 | 1.99 | 92.16 | 71.21 | 92.28 | 5.17 | 6.18 | 2.20 | 8.67 | 1.44 |
| OECD | 1.52 | -4.46 | 3.29 | 2.04 | 0.82 | 0.44 | 0.84 | 0.12 | 51.57 | 50.23 | 54.50 | 1.43 | 40.82 | 31.91 | 60.74 | 2.61 | 2.51 | 0.41 | 5.37 | 1.19 |
| Non-OECD | 1.11 | -3.17 | 6.27 | 2.14 | 0.74 | 0.56 | 0.78 | 0.18 | 58.42 | 55.23 | 60.09 | 2.42 | 38.91 | 35.40 | 49.19 | 3.12 | 4.08 | 3.53 | 9.06 | 2.18 |
| **Regional economies** | | | | | | | | | | | | | | | | | | | | |
| East Asia and Pacific | 4.91 | -0.76 | 7.61 | 1.69 | 0.71 | 0.63 | 0.78 | 0.16 | 43.40 | 41.77 | 47.32 | 1.63 | 41.49 | 27.61 | 71.48 | 1.14 | 2.36 | 1.43 | 3.21 | 0.50 |
| Europe and Central Asia | 1.41 | -5.54 | 3.88 | 2.42 | 0.73 | 0.59 | 0.76 | 0.02 | 49.52 | 47.46 | 53.90 | 2.03 | 97.99 | 87.73 | 98.02 | 6.87 | 3.91 | 1.15 | 8.89 | 2.37 |
| Latin America and Caribbean | 2.12 | -6.67 | 6.43 | 3.06 | 0.68 | 0.51 | 0.72 | 0.09 | 53.19 | 49.11 | 59.58 | 3.45 | 40.31 | 22.52 | 59.56 | 11.88 | 3.43 | 2.36 | 5.18 | 0.69 |
| MENA | 3.87 | 0.68 | 7.87 | 1.87 | 0.74 | 0.62 | 0.89 | 0.03 | 57.57 | 54.42 | 65.98 | 3.78 | 43.37 | 36.98 | 59.99 | 7.33 | 2.62 | 0.96 | 5.70 | 1.56 |
| North America | 1.78 | -3.55 | 3.85 | 1.89 | 0.79 | 0.49 | 0.92 | 0.07 | 50.33 | 48.97 | 53.58 | 1.42 | 83.96 | 59.50 | 83.99 | 6.69 | 1.77 | 1.00 | 2.93 | 0.61 |
| South Asia | 5.71 | -5.23 | 7.67 | 3.01 | 0.68 | 0.48 | 0.90 | 0.11 | 58.11 | 51.16 | 65.88 | 4.69 | 42.99 | 30.03 | 49.49 | 5.84 | 1.61 | 0.61 | 3.34 | 0.63 |
| Sub-Saharan Africa | 3.99 | -1.99 | 6.55 | 2.17 | 0.72 | 0.70 | 0.88 | 0.09 | 87.41 | 82.64 | 90.18 | 2.24 | 46.06 | 38.89 | 56.39 | 5.56 | 2.27 | 1.62 | 3.08 | 0.46 |
|  |  | | | |  | | | |  | | | |  | | | |  | | | |
| **Full panel** | 3.78 | 1.76 | 7.90 | 1.78 | 1.19 | 1.04 | 1.27 | 0.06 | 74.28 | 71.61 | 75.11 | 0.99 | 9.75 | 9.30 | 10.07 | 0.20 | 56.13 | 49.26 | 60.73 | 3.24 |
| **Income level economies** | | | | | | | | | | | | | | | | | | | | |
| Low-income | 5.92 | 2.61 | 11.01 | 2.39 | 2.63 | 2.58 | 2.74 | 0.05 | 19.90 | 19.41 | 20.55 | 0.47 | 10.90 | 8.92 | 9.20 | 0.10 | 53.09 | 46.45 | 60.41 | 5.03 |
| Middle-income | 4.87 | 2.44 | 9.32 | 2.11 | 1.17 | 0.98 | 1.28 | 0.07 | 35.23 | 34.12 | 36.59 | 0.71 | 9.81 | 9.31 | 10.25 | 0.28 | 51.74 | 43.49 | 60.61 | 5.59 |
| Upper middle-income | 4.50 | 1.35 | 9.13 | 2.10 | 0.72 | 0.47 | 0.83 | 0.08 | 48.41 | 47.57 | 48.99 | 0.42 | 9.85 | 9.76 | 9.95 | 0.62 | 51.92 | 43.74 | 62.05 | 0.37 |
| High-income | 4.88 | 1.48 | 9.52 | 1.38 | 1.03 | 0.69 | 1.18 | 0.16 | 90.33 | 76.15 | 91.37 | 2.17 | 8.15 | 6.99 | 10.14 | 1.00 | 68.02 | 50.33 | 68.98 | 5.15 |
| OECD | 2.07 | 1.06 | 3.13 | 0.64 | 0.68 | 0.51 | 0.78 | 0.08 | 87.80 | 87.26 | 88.52 | 0.35 | 9.94 | 9.87 | 10.02 | 0.33 | 52.14 | 43.88 | 56.93 | 4.28 |
| Non-OECD | 5.67 | 2.78 | 8.17 | 2.14 | 1.09 | 0.67 | 1.15 | 0.12 | 76.13 | 58.37 | 77.09 | 1.11 | 8.18 | 7.78 | 10.17 | 2.19 | 58.15 | 39.38 | 60.17 | 3.40 |
| **Regional economies** | | | | | | | | | | | | | | | | | | | | |
| East Asia and Pacific | 3.00 | 1.30 | 7.79 | 1.42 | 0.70 | 0.44 | 0.83 | 0.09 | 56.34 | 53.20 | 59.40 | 1.96 | 9.91 | 9.83 | 9.98 | 0.40 | 60.12 | 49.77 | 69.72 | 6.42 |
| Europe and Central Asia | 2.58 | 1.27 | 5.14 | 1.11 | 0.35 | 0.18 | 0.48 | 0.09 | 65.02 | 62.29 | 67.55 | 1.85 | 9.93 | 9.82 | 9.99 | 0.05 | 75.81 | 65.34 | 84.12 | 5.87 |
| Latin America and Caribbean | 4.15 | 2.18 | 7.61 | 1.73 | 1.11 | 0.91 | 1.37 | 0.17 | 51.02 | 48.65 | 52.58 | 1.21 | 9.76 | 9.68 | 9.86 | 0.28 | 45.25 | 41.34 | 48.14 | 1.86 |
| MENA | 6.19 | -5.74 | 17.34 | 6.16 | 2.00 | 1.77 | 2.18 | 0.12 | 45.65 | 42.85 | 49.20 | 1.98 | 9.40 | 9.10 | 9.69 | 0.19 | 82.58 | 72.01 | 91.69 | 5.19 |
| North America | 1.98 | 0.64 | 3.98 | 0.86 | 0.82 | 0.55 | 0.98 | 0.13 | 88.69 | 81.41 | 91.25 | 2.30 | 9.99 | 9.83 | 10.08 | 0.05 | 30.32 | 26.64 | 33.56 | 2.28 |
| South Asia | 6.04 | 3.11 | 10.68 | 2.11 | 1.43 | 1.14 | 1.82 | 0.21 | 36.74 | 32.62 | 39.24 | 1.88 | 7.99 | 6.81 | 8.03 | 0.05 | 42.78 | 30.86 | 52.79 | 6.57 |
| Sub-Saharan Africa | 5.44 | 2.67 | 10.53 | 2.38 | 2.71 | 2.62 | 2.76 | 0.04 | 29.86 | 28.77 | 31.21 | 0.68 | 8.21 | 5.76 | 8.56 | 0.03 | 54.00 | 44.97 | 63.29 | 5.44 |
| Notes: ECG = Economic growth, CFII – Composite financial inclusion index, ADR = Age dependency ratio, CRP = Credit to the private sector, FDI = Foreign direct investment, INF = Inflation rate, PGR = Population growth rate, RUL = Rule of law, SER = School enrollment ratio, TOP = Trade openness, OECD = Organization for economic cooperation and development, MENA = Middle-easter and north Africa. | | | | | | | | | | | | | | | | | | | | |
